# Single-centre observational study of the safety and efficacy of thoracoscopy under local anaesthesia for the management of thoracic infections

**DOI:** 10.1186/s13104-024-06794-9

**Published:** 2024-05-06

**Authors:** Kohei Fujita, Zentaro Saito, Takanori Ito, Takuma Imakita, Issei Oi, Osamu Kanai, Yuki Yamamoto, Hiroaki Hata, Satoru Sawai, Kiminobu Tanizawa

**Affiliations:** 1 Division of Respiratory Medicine, Center for Respiratory Diseases, National Hospital Organization Kyoto Medical Center, 1-1, Fukakusa-Mukaihata, Fushimi, Kyoto, Kyoto Japan; 2 HiLung Inc., Kyoto, Japan; 3 Department of Surgery, National Hospital Organization Kyoto Medical Center, Kyoto, Japan; 4 Department of Thoracic Surgery, National Hospital Organization Kyoto Medical Center, Kyoto, Japan

**Keywords:** Thoracoscopy, Local anaesthesia, Infections, Pyothorax, Empyema

## Abstract

**Objectives:**

Thoracoscopy under local anaesthesia is widely performed to diagnose malignancies and infectious diseases. However, few reports have described the use of this procedure for diagnosing and treating intrathoracic infections. This study aimed to evaluate the safety and efficacy of thoracoscopy under local anaesthesia for the management of intrathoracic infections.

**Results:**

Data from patients who underwent thoracoscopy procedures performed by chest physicians under local anaesthesia at our hospital between January 2018 and December 2023 were retrospectively reviewed. We analysed their demographic factors, reasons for the examinations, diseases targeted, examination lengths, anaesthetic methods used, diagnostic and treatment success rates, as well as any adverse events. Thirty patients were included. Of these, 12 (40%) had thoracoscopies to diagnose infections, and 18 (60%) had them to treat pyothorax. In terms of diagnosing pleurisy, the causative microorganism of origin was identified via thoracoscopy in only three of 12 (25.0%) patients. For diagnosing pyothorax, the causative microorganism was identified in 7 of 18 (38.9%) patients. Methicillin-resistant *Staphylococcus aureus* was the most common causative microorganism identified. The treatment success rates were very high, ranging between 94.4 and 100%, whereas the identification rate of the causative microorganisms behind infections was low, ranging between 25.0 and 38.9%. The most frequent adverse events included perioperative hypoxaemia and pain. There were two (6.7%) serious adverse events of grade ≥ 3, but none resulted in death.

**Conclusions:**

The efficacy of managing intrathoracic infections through thoracoscopy under local anaesthesia is commendable. Nonetheless, the diagnostic accuracy of the procedure, regarding the precise identification of the causative microorganisms responsible for intrathoracic infections, persists at a notably low level, presenting a substantial clinical hurdle.

## Introduction

Thoracoscopy has traditionally been performed under general anaesthesia; however, methods for performing the procedure under local anaesthesia have recently been established [1, 2], and many medical institutions in Japan are now performing thoracoscopies under local anaesthesia [3]. Because thoracoscopies can be performed under local anaesthesia, they are often performed by chest physicians. In addition to the diagnosis of thoracic malignancies, thoracoscopies can also be used for infectious diseases, such as in the treatments of infectious pleurisy and pyothorax [4].

As there are few reports on the use of thoracoscopy under local anaesthesia for the diagnosis and treatment of intrathoracic infections, we report our experience herein.

## Patients and methods

This single-centre, retrospective study was conducted at the National Hospital Organization (NHO) Kyoto Medical Center. We reviewed data from patients who underwent thoracoscopy under local anaesthesia between January 2018 and December 2023. Of these, we selected patients who underwent thoracoscopies performed by chest physicians for the diagnosis and treatment of infectious diseases. We excluded the patients whose treatment course could not be followed in the electronic medical record. The thoracoscopy under local anaesthesia was performed as follows. Insertion of the thoracoscope through the chest wall was performed under local anesthesia using lidocaine and midazolam, with a preoperative treatment of an intramuscular injection of pethidine or pentazine. The dosage of anesthetic drugs was left to the attending physician’s discretion. A pulse oximeter, blood pressure monitor, and electrocardiograph were worn during the examination to monitor the patients’ vital signs. The patient’s position was side-lying with the opposite side of the affected lesions. The position of the port placement was determined by a preoperative computed tomography scan and preoperative echographic findings. In the event of an emergency situation, the anaesthesiologist was asked to assist, and the patient was intubated, switched to ventilatory management and prepared to switch to ICU management immediately. In this study, the diagnosis of infectious pleurisy or pyothorax was based on the identification of a pathogenic microorganism or improvement achieved using chest drainage and antimicrobial treatment. Efficacy was assessed using the pathogenic microbiological diagnostic rate of biopsies and the success rate of treatments for pyothorax and pleurisy performed via thoracoscopy. Successful treatment of pyothorax and pleurisy was defined as an improvement in clinical symptoms and blood biochemistry findings (C-reactive protein [CRP] and white blood cell counts normalised or improved to near-normal levels) following thoracoscopy, and removal of the thoracic drain without subsequent relapse. The adverse events assessed were defined as follows. Hypoxaemia was defined as an oxygen saturation (SpO_2_) measurement of < 94% or a need for additional oxygen delivery. Hypertension was defined as systolic blood pressure > 160 mmHg. Hypotension was defined as systolic blood pressure < 80 mmHg. Fever was defined as a new onset of a fever of ≥ 38 °C following thoracoscopy. Pain was defined as any new pain that appeared after the procedure that required treatment. The severity of adverse events was assessed using the Common Terminology Criteria for Adverse Events (CTCAE) version 5.0. The observation period for complications was 3 months from the end of the examination. The NHO Kyoto Medical Center Review Board approved the study protocol (approval number: 23-051). The study is registered in the UMIN-Clinical Trials Registry as a retrospective study. The registration number is UMIN000053688.

## Results

We reviewed the data of 30 patients who underwent thoracoscopies under local anaesthesia performed by chest physicians, for the diagnosis and treatment of infectious diseases. The characteristics of the study participants and the statuses of their corresponding thoracoscopic procedures are shown in Table 1. The median age of the eligible participants was 74 years, they were predominantly male, and 73% had a history of smoking. Their most common comorbidities were hypertension (43.3%), diabetes mellitus (36.7%), and chronic obstructive pulmonary disease (COPD; 33.3%). Of the 30 patients, 12 (40%) underwent diagnostic thoracoscopies for infections and 18 (60%) had therapeutic thoracoscopies to treat pyothorax. Of the 18 patients treated for pyothorax, 16 were in stage 1, 2 in stage 2 and 0 in stage 3. The two stage 2 cases were both judged by the attending physician to have only partial organisation. Table 2 shows information on the perioperative management of thoracoscopic procedures. Firstly, sedation during the procedure, all patients were sedated with midazolam, in 83% of the patients were also sedated with pentazine. In the antibiotic administration, all patients for pyothorax treatment were given antibiotics, with sulbactam/ampicillin being the most common antibiotics. The median duration of drain insertion was 5.5 days for patients for diagnosis of infection and 9 days for patients for pyothorax treatment. The median duration of hospitalisation was 13 days for patients for the diagnosis of infection and 23 days for patients for pyothorax treatment. All patients for pyothorax treatment had septal thickening and required decortication and debridement. After decortication and debridement, thoracic cavities were irrigated.

**Table 1:** Patient characteristics

Patients’ characteristics	n = 30
Age, median	74 (26–85)
Sex (male)	27 (90)
BMI, mean	20.6 ± 3.0
Poor PS (≥ 2)	1 (3.3)
Smoking	22 (73.0)
Comorbidity	
COPD	10 (33.3)
Asthma	2 (6.7)
Cardiovascular	6 (20.0)
Hypertension	13 (43.30)
Diabetes mellitus	11 (36.7)
CKD/hemodialysis	7 (23.3)
Cerebrovascular	2 (6.7)
Malignancy	4 (13.3)
Purpose of thoracoscopy	
For diagnosis of infection	12 (40.0)
For pyothorax treatment	18 (60.0)
Stage 1 pyothorax	16
Stage 2 pyothorax	2
Stage 3 pyothorax	0

Data are shown in number (%)

*BMI* body mass index, *PS* performance status, *COPD* chronic obstructive pulmonary disease, *CKD* chronic kidney disease

**Table 2:** Information on the perioperative management of thoracoscopic procedures

	n = 30
Median time of procedure, min	44.5 (16–115)
Sedation	
Pethidine	3
Pentazine	25
Midazoram	30
Mean dose of midazoram, mg	6.27 ± 2.8
Antibiotics use during thoracoscopy	
For diagnosis of infection	1/12 (8.3%)
CTRX + VCM	1
For pyothorax treatment	18/18 (100%)
SBT/ABPC	13
MEPM	2
TAZ/PIPC	1
CTRX	1
TEIC	1
Median duration of drain removal, days	
For diagnosis of infection	5.5 (2–21)
For pyothorax treatment	9 (4–19)
Median duration of hospitalisation, days	
For diagnosis of infection	13 (4–56)
For pyothorax treatment	23 (14–58)

Data are shown in median (range), mean ± SD or number

*CTRX* ceftriaxone, *VCM* vancomycin, *SBT/ABPC* sulbactam/ampicillin, *MEPM* meropenem, *TAZ/PIPC* tazobactam/piperacillin, *TEIC* teicoplanin

**Fig. 1:**
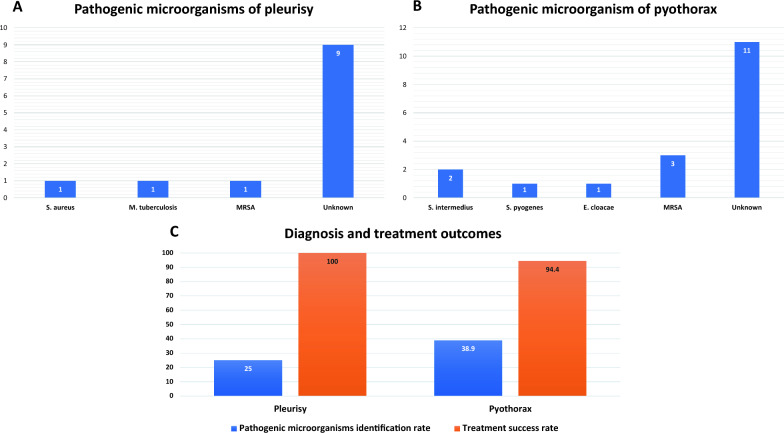
**A** shows the types and proportions of pathogenic microorganisms identified in pleurisy. **B** shows the types and proportions of pathogenic microorganisms identified in pyothorax. **C** shows the identification rate of pathogenic microorganisms and treatment success rate in pleurisy and pyothorax

Figure 1A and B show the types of pathogenic microorganisms identified in the cases of pleurisy and pyothorax. For pleurisy, the causative pathogenic microorganism was identified in only three (25.0%) of 12 patients. For pyothorax, the causative microorganism was identified in 7 (38.9%) of 18 patients. Methicillin-resistant *Staphylococcus aureus* (MRSA) was the most commonly identified pathogen responsible for the infections. Figure 1C shows the diagnostic and treatment success rates of pleurisy and pyothorax. The treatment success rates were quite high, ranging between 94.4 and 100%, whereas the successful diagnostic rate was low, falling between 25.0 and 38.9%. Table 3 shows the adverse events observed during the procedures. These mostly included perioperative hypoxaemia and pain. There were two (6.7%) serious adverse events of grade G3 or higher, but none resulted in death. Of 18 patients with pyothorax, one patient had a recurrence one month later, which was mild and treated with thoracic drainage only. Three patients were left with sequelae of mild pulmonary restriction. Of 12 patients with pleurisy, none of the patients had residual or recurrent pleurisy.

**Table 3:** Adverse events during thoracoscopy under local anaesthesia

	n = 30
Minimum SpO_2_, %	90 (84–98)
Maximum oxygenation (L/min)	3 (0–7)
Complication	
Pneumothorax	1 (3.3)
Hypoxia (SpO2 < 96%)	26 (86.7)
Hypertension (SBP ≥ 160 mmHg)	7 (23.3)
Hypotension (SBP < 80 mmHg)	2 (6.7)
Bleeding	0
Post-arrhythmia	0
Fever ≤ 38.0	5 (16.7)
Intubation	0
Infection/pneumonia	0
Perioperative pain	26 (86.7)
Prolonged poor arousal	3 (10.0)
CTCAE Grade ≥ 3	2 (6.7)
Death within 1 month	0

Data are shown in median (range) or number (%)

*SBP* systolic blood pressure, *CTCAE* Common Terminology Criteria for Adverse Events

## Discussion

The advantages of thoracoscopy under local anaesthesia are that it can be performed by chest physicians, can be performed in an endoscopy or procedure room rather than an operating theatre, and is relatively safe, simple, minimally invasive, and economically inexpensive. We found that the treatment success rate for infectious pleurisy and pyothorax using thoracoscopy under local anaesthesia was very high. However, its rate in terms of correctly identifying the causative pathogenic microorganisms behind thoracic infections was quite low, ranging between 25.0 and 38.9%. This study included only thoracoscopies performed by chest physicians; however, its outcomes were comparable to those of similar studies wherein the procedure was performed by thoracic surgeons [4].

In previous studies on tuberculous pleurisy, the rate of successful identification of the causative microorganism by thoracoscopy has been reported to be as low as 30–40% but also as high as 68.7% [5–9]. To the best of our knowledge, there are currently no reports in the literature on the use of thoracoscopy for diagnosing infections other than tuberculous pleurisy. In this study, we found that the procedure’s rate of successful diagnosis was even lower for other thoracic infections.

Despite the many benefits of thoracoscopy under local anaesthesia for tuberculous pleurisy [3], there is a paucity of data regarding the outcomes when it is used to treat infections such as pyothorax.

There are several restrictions regarding the treatment of pyothorax in Japan. The first is on the use of urokinase. Urokinase has thrombolytic properties and has been widely used in clinical practice; however, it is not covered by Japanese medical insurance and will be difficult to obtain after 2021 owing to an unstable supply chain. Another challenge with this treatment is that the tPA and DNase used in other countries are not approved in Japan and are, therefore, unavailable. This may make treatment with conventional thoracic drainage alone difficult necessitating more frequent use of thoracoscopy.

A previous report showed that thoracoscopy was associated with shorter hospital stays and reduced need for open drainage, compared to drainage alone [10]. Another study found that thoracoscopy reduced the median length of hospital stay, drainage duration, hospital cost, and occurrence of complications compared to drainage alone [11]. According to recent Japanese guidelines for pyothorax published by the Japanese Association for Chest Surgery [12], thoracoscopic intervention is also recommended for acute pyothorax.

Our current report revealed a low diagnostic success rate for the procedure despite recent advances in culture techniques. It is clinically beneficial that high treatment rates have been achieved for pyothorax, even when the causative microorganism has not been identified. However, clinicians should approach this option with some reservations. If the organism causing the infection cannot be identified, de-escalation of antimicrobials is often performed slowly and hesitantly in real-world clinical settings, and broad-spectrum antimicrobials must be used—which can also be problematic from the perspective of appropriate antimicrobial use.

The incidence rates of pyothorax and infectious pleurisy are expected to increase in Japan, owing to the country’s ageing population. Fujita et al. recently reported that thoracoscopy under local anaesthesia can be safely performed in older patients [13]. No more adverse events were observed in this study than in similar previous ones. These included two CTCAE Grade ≥ 3 events, neither of which resulted in death. Therefore, clinicians should consider performing thoracoscopy procedures more frequently if they are indicated, even in older patients.

The success rate of treating intrathoracic infections via thoracoscopy under local anaesthesia is high, and aggressive interventions are advisable in such cases. However, the diagnostic rate of the procedure, in terms of correctly identifying the causative microorganisms behind intrathoracic infections remains low, which remains a significant clinical challenge.

### Limitations

The study is a single-centre, retrospective study and may have significant bias in patient selection and treatment choice. In particular, the backup arrangements of thoracic surgeons are essential for chest physicians to perform thoracoscopy under local anaesthesia, and facilities with no or few thoracic surgeons are not adequately equipped to perform the examination. Furthermore, the present study was based on a small number of cases and cannot be immediately generalised. Small sample size could lead to size bias. Prospective studies with a larger number of patients in multicentre settings are needed.

## Data Availability

All data in this study can be provided with the permission of the Ethics Committee if there is a valid reason.
